# Comparison of shear bond strength and adhesive remnant index between precoated and conventionally bonded orthodontic brackets

**DOI:** 10.1186/2196-1042-14-39

**Published:** 2013-10-20

**Authors:** Ulises A Guzman, Laurance Jerrold, Peter S Vig, Ahmad Abdelkarim

**Affiliations:** 10435 Midtown Parkway, Jacksonville, FL 32246 USA; Orthodontic Consulting Group LLC, 9246 Sunrise Breeze Court, Jacksonville, FL 32256 USA; School of Orthodontics, Jacksonville University, 2800 University Blvd., N Jacksonville, FL 32211 USA; 2500 North State Street, Jackson, MS 39216-4505 USA; Department of Orthodontics, School of Dentistry, University of Mississippi, Jackson, MS USA

**Keywords:** Precoated brackets, Shear bond strength, Adhesive remnant index

## Abstract

**Background:**

The purpose of this study was to compare the shear bond strength and adhesive remnant index (ARI) at the enamel-bonding interface of precoated and conventionally bonded brackets, utilizing standardized procedures.

**Methods:**

The test sample consisted of 90 recently extracted bovine permanent mandibular incisors. The teeth were bonded using the same protocol and were tested in three different situations. A material testing systems machine was utilized for debonding, and the remaining adhesive on the tooth was recorded.

**Results:**

Immediately after bonding, we found that the shear bond strength of the precoated brackets (6.27 MPa) was significantly higher than that of conventional brackets (5.37 MPa) (*p* < 0.05). However, no significant differences in bond strength were found between the two bracket systems after 24 h of bonding or after thermocycling. The conventional brackets had higher ARI scores than the precoated bracket systems immediately after bonding and after 24 h.

**Conclusions:**

Since there were no significant differences in the bonding strength after 24 h, the immediate bonding strength of the precoated brackets during the first day does not appear to be a major advantage over the conventional bracket systems. However, less adhesive on the tooth after debonding is an advantage of precoated brackets.

## Background

The study and evaluation of the adhesive potential of a specific bonding system are complicated, as there are multiple variables that can influence the survival or longevity of the bracket-enamel interphase [1]. The two primary tests used for evaluating the strength of the orthodontic adhesives measure shear and tensile bond strengths. In the shear test, the force is directed parallel to the long axis of the tooth and as closely as possible to the bracket-tooth interface [2–4]. *In vitro* studies have shown that orthodontic brackets must be able to sustain loads from 5.9 to 7.8 mega-Pascals (MPa) of shear bond strength (SBS) to be considered clinically successful for orthodontic purposes [5].

There are many factors that can cause bond failure of orthodontic brackets, including the multifactorial nature of the oral environment which causes pH fluctuations, as well as the complex cyclic loading of chewing, alcohol-containing fluids, temperature variations, and food consistency, all of which make it difficult to specifically determine the reasons for failure [6–9]. When considering each of these factors, the true effectiveness and performance of any particular bracket-bonding system in *in vitro* studies become questionable when different studies are compared. However, if studies are performed under standardized testing conditions, they may generate more reliable information that may be useful in future studies.

To date, no standardization of these studies exists, and findings from individual studies have been inconsistent. Consequently, studies cannot be compared to each other if different methodologies, test fixtures, test substrates, and brackets are used [2]. Although *in vitro* studies should provide information that can be extrapolated to clinical practice, many of the factors involved hinder a true simulation of intraoral conditions [2–4, 10–12]. Moreover, many valuable bonding studies have been conducted utilizing different test substrates, human teeth, bovine teeth, and artificial materials such as porous ceramics under incomparable testing conditions with different methodologies and protocols, resulting in inconsistent information [13–19].

Numerous studies have made suggestions to overcome the problems associated with the clinical applicability of results from *in vitro* studies. Technical specifications, as described in ISO/TS 11405:2003, provide guidance for the selection of substrates and storage and handling conditions, as well as the essential characteristics of different test methods for quality testing. Therefore, the purpose of this study was to compare the SBS and adhesive remnant index (ARI) at the enamel-bonding interface between precoated and conventionally bonded brackets, utilizing standardized procedures, thereby facilitating comparisons among studies.

## Methods

### Study population

The test samples consisted of 90 recently extracted (<6 months) bovine permanent mandibular incisors. Bovine mandibular incisors are considered a viable option in bonding studies as they are readily available, inexpensive, and similar to human teeth. In addition, they have larger crystal grains and more lattice defects than human teeth, resulting in lower critical surface tension, probably related to their slightly lower bonding values than human teeth. Studies have demonstrated that bonding strength increases in older teeth as opposed to recently extracted teeth [20, 21].

These recently extracted incisors were obtained from the Animal Technologies, Inc. (Tyler, TX, USA). In order to standardize the study, we controlled for the variability of results by using one specific sample type; thus, deciduous mandibular incisors were excluded from the study. As reported by Oesterle et al., there are differences in bond strength between bovine deciduous incisors (21%) and permanent incisors (35%) [22]. The teeth were only obtained from The United States Department of Agriculture or equivalent inspected facilities, where animals received ante- and post-mortem inspection, and were free of contagious diseases. The substrate was collected from the animals < 30 months from the same lot. The bovine teeth were extracted from a different lot, representing different extraction times, to standardize and control for the variability of results, as reported by Nakamichi et al. who established that bonding strength increases as teeth age [20].

### Testing conditions

Immediately after extraction, the teeth were washed in running water, and all blood and adherent tissue were removed. The teeth were then placed in distilled water and stored at 37°C. The 90 teeth were divided into three groups of 30 specimens; the three groups represented three time points: T1, T2, and T3. A standard reproducible flat surface was utilized on each tooth, where two brackets (precoated and conventionally bonded) were placed on each facial surface. The tooth surfaces were kept wet at all times. The enamel was cleaned with pumice. The enamel was etched with 35% phosphoric acid gel (3 M Unitek, Monrovia, CA, USA) for 20 s, rinsed under running water for 20 s, and then dried with oil- and moisture-free compressed air. The teeth were mounted in a custom-made baseholder and then bonded (Transbond™ XT, 3 M Unitek) and light cured using Ortholux LED (3 M Unitek) at a wavelength of 460 nm for a total of 10 s (5 s on the mesial and 5 s on the distal aspects) on selected brackets (maxillary left incisors). This was performed in a standardized manner, utilizing height gauge with an identical amount of pressure applied to each bracket, namely, 30 g of force using a force gauge (Dontrix gauge, Invecta®, GAC, Bohemia, NY, USA).

The following bracket systems were used: type A was Smart Clip MBT High TQ (3 M Unitek) self-ligating metal brackets, and type B was APC™ II Adhesive Coated Appliance Smart Clip MBT High TQ (3 M Unitek) self-ligating metal brackets. Each bracket system was tested at three different time points: (1) Very short-term (T1): 15 min after bonding, (2) short-term (T2): 24 h after bonding, and (3) after thermocycling (T3): 1,000 cycles in water between 5°C and 55°C after 24 h of storage in water at 37°C. Each cycle was at least 20 s, with a transfer time between baths for 5 to 10 s.

### Testing of shear bond strength

The specimens were stored in distilled water prior to testing at (37°C ± 2°C) and tested immediately after removal from water. An MTS machine (MTS Insight 1, MTS Systems Corporation, Eden Prairie, MN, USA) was used to evaluate the force applied to debond the brackets. Debonding was performed with an MTS Insight 1 machine with a blade design, pin under an occlusogingival load at a crosshead speed of 0.5 mm/min, beginning at 2 mm from the bracket to the metal pin of the MTS unit that recorded the test results. The results were recorded in MPa by a computer connected to the machine. Each tooth was oriented so that its facial surface was parallel to the direction of force during the shear testing. The shear force application was directly applied to the bracket-tooth interface, near the base.

### Testing the adhesive remnant index

After bracket failure, the enamel surface was examined under optical magnification (×10), and the amount of adhesive remaining on the tooth was recorded using the ARI. The criteria for ARI scoring were as follows: 0, no adhesive on the tooth; 1, less than 50% adhesive on the tooth; 2, more than 50% adhesive on the tooth; and 3, all adhesive remained on the tooth.

### Statistical analyses

Student's *t* test analysis was used to determine whether there was a significant difference in the shear bond strength between the two test groups. One-factor analysis of variance (ANOVA) was used to analyze the difference and to compare each bracket performance within itself at T1, T2, and T3. Mann–Whitney non-parametric statistical analysis was used to compare the ARI between the two test groups, and Kruskal-Wallis non-parametric statistical analysis was used to compare each bracket performance within itself at T1, T2, and T3. All the statistical analyses were performed using the SPSS software (version 18, SPSS, Inc., Chicago, IL, USA). *P* values less than 0.05 were considered significant.

## Results

### Shear bond strength

The SBS values for the two bonding systems are presented in Tables 1 and 2. The results demonstrated that immediately after bonding, the mean SBS for the precoated bracket system was significantly higher than that in the conventional group (6.27 and 5.37 MPa, respectively, *p* < 0.05). However, no significant difference was found in the SBS between both groups at 24 h and after thermocycling. When comparing the mean SBS difference within the conventional bracket system at different time points (T1, T2, and T3), the ANOVA analysis demonstrated that the mean SBS difference was significantly higher after 24 h compared to immediately after bonding. Comparing the SBS mean difference within the precoated bracket system within the same bracket system at T1, T2, and T3, the ANOVA analysis demonstrated a significantly higher mean value after 24 h compared to immediately after bonding.

**Table 1:** **Mean (SD) of shear bond SBS measured in MPa**

Time point	Conventional brackets SBS	Precoated brackets SBS	***P*** value
T1 (immediately after)	5.37 (1.62)	6.27 (1.43)	0.028
T2 (24 h after)	6.82 (2.24)	7.19 (2.13)	0.516
T3 (after thermocycling)	6.54 (3.08)	7.39 (2.36)	0.238

**Table 2:** **Mean (SD) of shear bond strength differences between the different time points**

Time point difference	Conventional brackets SBS difference	***P*** value	Precoated brackets SBS difference	***P*** value
T2 (24 h after) vs.T1 (immediately after)	1.45 (0.62)	0.021	1.32 (1.00)	0.017
T3 (after thermocycling) vs. T2 (24 h after)	−0.28 (0.84)	0.656	0.2 (0.23)	0.715
T3 (after thermocycling) vs. T1 (immediately after)	1.17 (1.46)	0.061	1.12 (0.93)	0.051

### Adhesive remnant index

The results of the ARI analyses are presented in Tables 3 and 4. The significant differences were found in the ARI scores between the conventional and the precoated bonding systems when tested immediately and 24 h after bonding (*p* < 0.05). When comparing the ARI within the same bracket system in different time points (T1, T2, and T3), there was insufficient evidence to conclude that there was a significant difference in the mean ARI scores for both bracket groups. As Table 5 demonstrates, significantly higher ARI scores were found in the conventional group, which means that most adhesive remained on teeth after debonding of the conventional brackets.

**Table 3:** **Mean (SD) of ARI at T1 to T3**

Time point	Conventional brackets ARI	Precoated brackets ARI	***P*** value
T1 (immediately after)	1.97 (0.81)	1.47 (0.90)	0.027
T2 (24 h after)	1.73 (0.79)	1.30 (0.84)	0.043
T3 (after thermocycling)	1.67 (0.92)	1.40 (0.93)	0.270

**Table 4:** **Mean (SD) of adhesive remnant index differences between the different time points**

Time point difference	Conventional brackets ARI difference	***P*** value	Precoated brackets ARI difference	***P*** value
T2 (24 h after) vs. T1 (immediately after)	−0.24 (0.02)	0.767	−0.17 (0.06)	0.782
T3 (after thermocycling) vs. T2 (24 h after)	−0.06 (0.13)	0.817	0.1 (0.09)	0.798
T3 (after thermocycling) vs. T1 (immediately after)	−0.3 (0.11)	0.656	−0.07 (0.03)	0.815

**Table 5:** **ARI scores**

	Score	Conventional brackets (%)	Precoated brackets (%)	Chi-square	***P*** value
Immediately after bonding	0	0 (0)	5 (17)	4.600	0.032
	1	10 (33)	10 (33)		
	2	11 (37)	12 (40)		
	3	9 (30)	3 (10)		
24 h after bonding	0	1 (3)	5 (17)	4.117	0.042
	1	11 (37)	13 (43)		
	2	13 (40)	10 (33)		
	3	6 (20)	2 (7)		
After thermocycling	0	1 (3)	5 (17)	4.117	0.042
	1	11 (37)	13 (43)		
	2	13 (40)	10 (33)		
	3	6 (20)	2 (7)		

0, no adhesive on the tooth; 1, less than 50% adhesive on the tooth; 2, more than 50% adhesive on the tooth, and 3, all adhesive remained on the tooth.

## Discussion

The primary goal of this study was to follow the technical specifications established and recommended by ISO/TS 11405:2003. The standardization of the *in vitro* studies allows better comparison among studies, and the resulting information may provide relevant clinical information that can influence orthodontic treatment decisions. Determining the levels of clinically accepted bond strength and the best bracket system in terms of efficiency, cost, and bonding predictability have been and will continue to be of special interest.

In this study, it was found that the SBS of the precoated bracket and bonding system was significantly higher than that of the conventional bracket system immediately after bonding. However, no differences in bond strength were found 24 h after bonding or after thermocycling, confirming the exponential increase in bond strength the first few minutes after treatment and a gradual increase in bond strength after the first 24 h, as previously reported by Braem et al. [19]. The ANOVA analysis, which was performed to determine any differences in bond strength within the same bracket system, demonstrated that the mean SBS of the conventional bracket system after 24 h was higher than that immediately after bonding.

The conventional bracket demonstrated higher ARI scores than the precoated bracket system immediately after bonding and 24 h after bonding. In other words, the precoated brackets had less adhesive remaining after debonding. This is perhaps due to the fact that precoated brackets have a premeasured uniform layer of adhesive that is coated in a manner that leaves less adhesive after application, perhaps facilitating optimal application of the brackets to the tooth surface, minimizing adhesive quantity. It is also possible that this is a result of the uniform pressure applied in placing the adhesive on the mesh during machine precoating of the bracket during manufacturing, allowing better penetration of the bracket mesh.

When extrapolating these results to a clinical setting, it can be concluded that better bonding strength may not be a major advantage of the precoated brackets over the conventional ones. The time at which both bracket systems revealed possible clinical significance is usually addressed by recommending to orthodontic patients a soft diet during the first 24 h after bonding. However, less adhesive remnant is an advantage of precoated brackets. Other advantages of precoated brackets cannot be denied, such as consistent quality and quantity of the adhesive, eliminating the need of adhesive application, reducing waste, allowing easier clean-up, and improving asepsis.

## Conclusions

This study allows the following conclusions to be made:

1. Compared to the conventional bracket system, the precoated bracket systems have significantly higher SBS only immediately after bonding. This may not have clinical significance, especially if the patient is instructed to start a soft diet if the conventional bracket system is used.
2. No difference in bond strength exists between precoated and conventional brackets 24 h after bonding or after thermocycling, confirming the exponential increase in bond strength after cure and the gradual increase in bond strength in the first 24 h.
3. When compared to conventional brackets, precoated brackets leave less adhesive remnant.
4. Immediate bond strength may not be a major advantage of one bracket over another between precoated and conventional bracket systems; however, less adhesive on the tooth after debonding is an advantage of precoated brackets.

## References

[1] Leloup G, D'Hoore W, Bouter D, Degrange M, Vreven J (2001). Meta-analytical review of factors involved in dentin adherence. J Dent Res.

[2] Swartz ML (2007). Limitations of in vitro orthodontic bond strength testing. J Clin Orthod.

[3] Mandall NA, Millett DT, Mattick CR, Hickman J, Worthington HV, Macfarlane TV (2002). Orthodontic adhesives: a systematic review. J Orthod.

[4] Fox NA, McCabe JF, Buckley JG (1994). A critique of bond strength testing in orthodontics. Br J Orthod.

[5] Reynolds I (1975). A review of direct orthodontic bonding. Br J Orthod.

[6] Arhun N, Arman A, Sesen C, Karabulut E, Korkmaz Y, Gokalp S (2006). Shear bond strength of orthodontic brackets with 3 self-etch adhesives. Am J Orthod Dentofacial Orthop.

[7] Eliades T, Bourauel C (2005). Intraoral aging of orthodontic materials: the picture we miss and its clinical relevance. Am J Orthod Dentofacial Orthop.

[8] Millett DT, Hallgren A, Cattanach D, McFadzean R, Pattison J, Robertson M, Love J (1998). A 5-year clinical review of bond failure with a light-cured resin adhesive. Angle Orthod.

[9] De Saeytijd C, Carels CE, Lesaffre E (1994). An evaluation of a light-curing composite for bracket placement. Eur J Orthod.

[10] Eliades T, Brantley WA (2000). The inappropriateness of conventional orthodontic bond strength assessment protocols. Eur J Orthod.

[11] Murray SD, Hobson RS (2003). Comparison of in-vivo and in-vitro shear bond strength. Am J Orthod Dentofacial Orthop.

[12] Sunna S, Rock WP (1998). Clinical performance of orthodontic brackets and adhesive systems: a randomized clinical trial. Br J Orthod.

[13] Finnema KJ, Ozcan M, Post WJ, Ren Y, Dijkstra PU (2010). In-vitro orthodontic bond strength testing: a systematic review and meta-analysis. Am J Orthod Dentofacial Orthop.

[14] Yi GK, Dunn WJ, Taloumis LJ (2003). Shear bond strength comparison between direct and indirect bonded orthodontic brackets. Am J Orthod Dentofacial Orthop.

[15] Kula K, Schreiner R, Brown J, Glaros A (2002). Clinical bond failure of pre coated and operator coated orthodontic brackets. Orthod Craniofac Res.

[16] Bishara SE, Olsen M, Von Wald L (1997). Comparisons of shear bond strength of precoated and uncoated brackets. Am J Orthod Dentofacial Orthop.

[17] Cooper RB, Goss M, Hamula W (1992). Direct bonding with light-cured adhesive precoated brackets. J Clin Orthod.

[18] Oliver RG (1988). The effect of different methods of bracket removal on the amount of residual adhesive. Am J Orthod Dentofacial Orthop.

[19] Braem M, Lambrechts P, Vanherle G, Davidson CL (1987). Stiffness increase during the setting of dental composite resins. J Dent Res.

[20] Nakamichi I, Iwaku M, Fusayama T (1983). Bovine teeth as possible substitutes in the adhesion test. J Dent Res.

[21] Moriwaki Y, Kani T, Kozatani T, Tsutsumi S, Shimode N, Yamaga R (1968). The crystallinity change of bovine enamel during maturation. Jpn J Dent Mat.

[22] Oesterle LJ, Shellhart WC, Belanger GK (1998). The use of bovine enamel in bonding studies. Am J Orthod Dentofacial Orthop.

